# Bigger is not always better: Viability selection on body mass varies across life stages in a hibernating mammal

**DOI:** 10.1002/ece3.7304

**Published:** 2021-03-09

**Authors:** Alexandra H. M. Jebb, Daniel T. Blumstein, Pierre Bize, Julien G. A. Martin

**Affiliations:** ^1^ School of Biological Sciences University of Aberdeen Aberdeen UK; ^2^ The Rocky Mountain Biological Laboratory Crested Butte CO USA; ^3^ Department of Ecology and Evolutionary Biology University of California Los Angeles CA USA; ^4^ Department of Biology University of Ottawa Ottawa ON Canada

**Keywords:** age‐dependency, body mass, maximum running speed, phenotypic selection, sexual selection, viability selection

## Abstract

Body mass is often viewed as a proxy of past access to resources and of future survival and reproductive success. Links between body mass and survival or reproduction are, however, likely to differ between age classes and sexes. Remarkably, this is rarely taken into account in selection analyses. Selection on body mass is likely to be the primary target accounting for juvenile survival until reproduction but may weaken after recruitment. Males and females also often differ in how they use resources for reproduction and survival. Using a long‐term study on body mass and annual survival in yellow‐bellied marmots (*Marmota flaviventer*), we show that body mass was under stabilizing viability selection in the first years of life, before recruitment, which changed to positive directional selection as age increased and animals matured. We found no evidence that viability selection across age classes on body mass differed between sexes. By investigating the link between running speed and body mass, we show that the capacity to escape predators was not consistent across age classes and followed a quadratic relationship at young ages only. Overall, our results indicate that mature age classes exhibit traditional patterns of positive viability selection on body mass, as expected in a hibernating mammal, but that mass in the first years of life is subjected to stabilizing selection which may come from additional predation pressures that negate the benefits of the largest body masses. Our study highlights the importance to disentangle selection pressures on traits across critical age (or life) classes.

## Graphical abstract

Age‐specific links between body mass and survival are rarely taken into account in selection analyses. In our study, we show how selection on body mass changes with age in a hibernating mammal, yellow‐bellied marmots (*Marmota flaviventer*), from stabilizing selection at young ages to directional at older. We also show that predation on young individuals may provide a mechanism by which selection acts by quantifying the relationship between maximum running speed and body mass at all ages

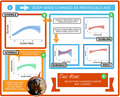

## 1 INTRODUCTION

Body mass is considered a key life‐history trait in many taxa (Roff, 2001) since it not only reflects past and present access to resources but it is also an important determinant of survival and reproductive success (Kingsolver & Pfennig, 2004). However, the relationship between fitness and body size may be hard to determine. The co‐variance between mass, or size, and fitness can differ among ontogenetic stages (e.g., juvenile vs. adult viability) or continuously with age such that the selective pressure changes over an individual's lifespan. Groups of individuals presenting traits at extreme ends of a distribution might be eliminated before recruiting into the population (Mojica & Kelly, 2010), creating “invisible fractions” (sensu Grafen, 1988) whose traits are never measured or detected. By ignoring the existence of multiple stages, selection analyses may fail to account for important selective events that could potentially change the direction of selection entirely. Most studies addressing selection on body mass are, however, restricted to one or two life stages (Pelletier et al., 2007; Barbraud et al., 2003). To gain insight on selection on body mass and possible conflicting effects of the “invisible fraction” on measures of selection, an alternative approach might be to examine selection independently across more specific groupings; defined by the varying pressures acting upon different age classes (Wilson et al., 2005).

Animal lifespans can be easily divided into at least three stages: growth and early development; reproduction and adulthood; and, ultimately, senescence and end of life. For neonates and juveniles, where the emphasis in the earliest life stages is on rapid growth including fat and somatic gain (Campbell & Dobson, 1992), the expectation is that selection would favor large, fast‐growing individuals who are precocial at an earlier age. Mass, at these ages, is important for survival, and recruitment into the population, in the following years (Both et al., 1999). Upon recruitment and attainment of adulthood, reproduction becomes a key pressure‐regulating body mass. Females often depend on body mass to be more fecund (Derocher & Stirling, 1994; Nespolo & Bacigalupe, 2009), more dominant (Huang et al., 2011), and better able to bear the costs of parental care (e.g., lactation, Kroeger et al., 2018). In males, larger body mass can be key to winning male–male competition (Haley et al., 1994) and defending higher quality territories (Candolin & Voigt, 2001). This is either because it enables males to survive prolonged periods of starvation during the breeding season (Haley et al., 1994) or because it increases competitive advantage and enables males to achieve higher social rank (Huang et al., 2011; Pelletier & Festa‐Bianchet, 2006). Furthermore, preferential mating of individuals in one sex group for specific body masses, or for traits that are closely associated with body mass (e.g., female preference for loud roars in male *Cervus elaphus*; Charlton et al., 2007), can create strong biases towards certain trait values. Such sexual selection is a powerful force acting on many traits in nature (Kingsolver et al., 2001). The high incidence of polygyny, in mammals, creates strong selective pressures that promote sexual dimorphism in body mass (Weckerly, 1998), often reflective of the different roles played by the different sexes. Ultimately, differences in selection pressures between males and females are expected to lead to the evolution of adult males that are often much larger than females (Weckerly, 1998). Finally, as age advances, condition‐dependent traits like body mass are expected to show accelerating declines towards the point of death (as reviewed in Nussey et al., 2013). Many studies conducted across the lifespan of individuals have shown the effect of senescence on body mass (Douhard et al., 2017; Kroeger et al., 2018; Nussey et al., 2017). This may act via the combined negative effects of physiological declines such as sarcopenia (Janssen et al., 2000) as well as age‐related changes in metabolism and satiety hormones (Wilson & Morley, 2003).

There are known constraints and costs associated with attaining larger body mass including increased basal metabolic rate, decreased locomotor performance, and obesity‐related diseases. In addition, predation and environmental conditions can strongly impact body mass variation and selection. Climatic effects, like ambient temperature, can increase metabolic rates and the energy expenditure of individuals (Humphries et al., 2005), and the impact of such conditions can be specific to life stages. For example, gestating female rats (*Rattus rattus*) appear to reduce body mass to cope with the energetic demands of reproduction under cold temperatures (Luz & Griggio, 1992). Additionally, selective predation (Taylor & Cox, 2019) of individuals of a set age category, sex, or size could completely alter the mean body mass within a population. Running fast to escape predation has a high energy cost (Hall et al., 2004) which may prevent the accumulation of large energy reserves as body mass. Furthermore, fat storage has the potential to incur large locomotor costs resulting in an increased risk of predation (Witter & Cuthill, 1993). If individuals must move quickly to evade predation, their capacity to survive under this circumstance may depend on attaining an optimal body mass with respect to speed of escape (Garland, 2009). Accordingly, in hibernating Belding's ground squirrels (*Urocitellus beldingi*), the fattest individuals are the slowest ones during the active season and thus selection favors delaying the onset of hibernation body mass (Trombulak, 1989).

Here, we investigate stage‐ and sex‐dependent selection on a hibernating sciurid rodent: the yellow‐bellied marmot *(Marmota flaviventer)*. Hibernators are, perhaps, among the species which are most dependent on body mass for survival and so provide a good model for examining the effects of selection on this trait. The major over‐winter mortality of hibernating species arises when the energetic requirements of hibernation exceed the reserves gained before immergence (as reviewed in Humphries et al., 2003). In species which survive long winters using body reserves, this is expected to create a strong selective pressure to be large and store fat to maximize survival probability. Yellow‐bellied marmots depend largely on body reserves accumulated during the summer season for survival over the harsh winter season and, as a consequence, they undergo strong circannual fluctuations in mass where they can more than double their mass during the summer (Armitage, 2014). However, these animals also exhibit growth and body mass changes at different life stages, supporting the idea that the selective pressures regulating this trait may be stage‐dependent. Individuals in their first, second, and third year (females only) of life exhibit rapid growth and, particularly at the youngest ages, need to achieve both somatic growth and accumulate energy reserves (Heissenberger et al., 2020). Individuals in their second year of life may disperse (Schwartz et al., 1998) and at older ages, individuals must allocate energy resources to both maintenance and reproduction (Armitage, 2014). At elderly ages, individuals exhibit a significant senescence in body mass (Kroeger et al., 2018). Additionally, the relationship between body mass before hibernation, growth rate, and annual survival of females vary with age (Heissenberger et al., 2020), further evidencing that changes in selection could be expected across age classes.

Yellow‐bellied marmots also exhibit consistent sexual dimorphism in body mass from the time of weaning so that males are consistently larger than females (Armitage, 2014). This relates directly to the reproductive strategy each sex employs: Males defend multiple females at set territories, gaining more reproductive success when highly aggressive (Olson & Blumstein, 2010; Wey & Blumstein, 2012) whereas females recruit female offspring into their own territories, gaining more reproductive success from being somewhat affiliative (Armitage, 1991; Schwartz et al., 1998; Wey & Blumstein, 2012). Furthermore, there are known disparities between survivals within the sexes, with females living longer lives (Schwartz et al., 1998).

Using data on body mass and annual survival from a long‐term study of yellow‐bellied marmots in Colorado's Rocky Mountains, we tested three hypotheses relating to viability selection on body mass. First, we hypothesized that the nature of selection acting on body mass changes across a lifespan and will be stage‐dependent. In marmots, body fat reserves are essential for ensuring over‐winter survival (Cordes et al., 2020). Consequently, we predicted that body mass should be under strong positive selection but that the viability selection on body mass should be stronger in the early stages and then weaken due to the removal from the population of extreme phenotypes (invisible selection effect, sensu Grafen). Second, we hypothesized that viability selection on body mass differs between the sexes due to sexual dimorphism and sexual selection. We predicted that viability selection on mass should be stronger on males across all ages compared with females. Finally, we hypothesized that external pressures, like predation or environmental harshness, may prevent attainment of excessively large body masses. We tested this last hypothesis in two ways. First, we tested whether the environmental harshness of the location of the marmot colony modified the relationship between body mass and survival. Marmots in the study population are distributed between spatially distinct colonies. Colonies are further divided into two different parts of the valley (up‐valley and down‐valley), with an elevational difference of (approximately) 300 m (Armitage, 2014). Differences in elevation within this site are closely associated with differences in phenology; snowmelt at the onset of the season is significantly later at higher elevation sites compared with lower sites and emergence of social groups is delayed by 14 days at higher elevations (Blumstein et al., 2004; Inouye et al., 2000). Second, we quantified whether larger masses impede running speed and, thus, escapes from attacks by predators. Over the summer, marmots undergo large increases in body mass to compensate for expected losses of up to 50% of their mass during hibernation (Armitage et al., 1976) and the addition of this extra mass might impede their capability to escape predators. If so, predation on individuals with the largest body masses may create stabilizing selection on mass before hibernation. We expected that the relationship between body mass and running speed would be quadratic, as initial increases in size may result in enhanced muscular or skeletal gain but that extreme fat gain at the largest mass values will limit maximum running speed. We expected, also, that age‐related variation in selection will be reflected by variation in the relationship between body mass and speed at different life stages, as similar pressures are expected to regulate maximum running speed.

## 2 MATERIALS AND METHODS

### 2.1 Study population and general methods

We used long‐term data collected between 1962 and 2018 from a resident population of yellow‐bellied marmots at the Rocky Mountain Biological Laboratory (RMBL) in Gothic, Colorado, USA. Every year, since 1962, individuals have been trapped during the active season (May–September). Trapping occurs at each colony on a fortnightly basis, using Tomahawk live traps (81 × 25 × 30 cm, Tomahawk Live Trap Co.). Upon their first capture, animals are identified using numbered ear‐tags (1005–3 Monel self‐piercing fish tags) and with unique nontoxic, dyed fur marks. At each trapping event, individuals are sexed and weighed, initially using a spring scale (to the nearest 50 g) and now using a digital balance (to the nearest 10 g). For individuals captured at the site, more than 90% of individuals are of known age because they were first trapped within their first or second year of life (Armitage et al., 1976). However, the population is not closed and few individuals of unknown age do immigrate into the system. Furthermore, some individuals who were born within the system emigrate out and are never trapped again; this is common in one‐year‐old marmots who tend to disperse around the time that the current years pups emerge but extremely rare otherwise (Blumstein et al., 2009; Montero et al., 2020).

For the individuals examined in this study, average age was 1.4 (0.8 for males and 1.9 for females), illustrating the fact that marmots have high mortality in early‐life, and the maximal age was 14 (11 for males and 14 for females). For analysis, four age‐categories were considered: juveniles (0 years), yearlings (1 year), subadults (2 years), and adults (3+ years). This classification is based on the different energy requirements at each life stage and reflects the trade‐offs between growth, physiological maintenance, and reproduction associated with each age class (Salsbury & Armitage, 2003). Juveniles experience the greatest growth (Armitage et al., 1976), yearlings, and subadults slightly reduced rates of somatic growth, whilst adults are assumed to have reached their maximal skeletal size (Armitage, 2014). From 2 years of age, reproduction is possible but the vast majority of marmots start to reproduce at age 3 or after (females: Ozgul et al., 2007; Schwartz et al., 1998). Age at first reproduction does not seem to be related to body mass and is mainly affected by reproductive suppression (Armitage, 2014). Maximum skeletal size is attained by age 4 (Armitage, 2014; Martin et al., 2013). However, for females, maximal August mass is attained at around 7–9 years of age, before a decrease in body mass occurs with age (Kroeger et al., 2018).

### 2.2 Maximum running speed

After each trapping event from late July onwards, we estimated marmot maximum running speed by making loud noises upon release from the handling bag to encourage marmots to run to their burrow (for further justifications and more detailed methods see Blumstein, 1992; Blumstein, Runyan, et al., 2004). Individuals are released at a distance from their burrow (mean = 11.59 m; maximum = 50.30 m) and only measures from individuals that ran in a straight line, across a homogeneous substrate, and incline, towards their burrow were retained for the analysis (Blumstein, 1992). Time needed to reach the burrow was recorded using a digital stopwatch, distance with a standard tape measure, and incline with a clinometer. Substrate was assigned to four different categories based on expected difficulty of the terrain, which is known to impact marmot running speed (Blumstein, Runyan, et al., 2004): dirt (no vegetation or stone), stone (uneven rocky surface or talus), low vegetation (below a running marmot's shoulders), and high vegetation (above a running marmot's shoulders). Maximum running speed was calculated as distance traveled/time (m/s). Previous studies evaluating maximum running speed in yellow‐bellied marmots removed runs shorter than 1.5 s to minimize measurement errors (Blumstein, 1992; Blumstein et al., 2010; Blumstein, Runyan, et al., 2004). For the purposes of this paper, we included all runs to avoid removing potentially biologically important observations. We justified this by surmising that if the weakest/poorest runners not only run slowly but also for a short time, then excluding short runs would remove important observations from the analysis. However, we also conducted a separate analysis with these individuals removed, the details of which can be found in the Appendix S1 (Appended Analysis and Table S3).

### 2.3 Prehibernation body mass

The body mass of *M. flaviventer* decreases over the course of hibernation, reaching the lowest point around emergence from the burrow (Armitage, 2014), and increases over the active, feeding season (Salsbury & Armitage, 2003). To capture this variation, each individual was weighed an average of 3.42 and maximally 25 times a year, across the active season. Due to large variation in the timing of the mass measurements, we estimated a body mass on 15 August for all individuals and used it as prehibernation body mass. By this date, individual body mass has begun to plateau, and consequently, this measure reflects annual growth. To do so, we fitted linear mixed‐effect models of body mass with linear and quadratic effects of day of the year, random intercepts for colonies, year, individual identity, and day of the year, as well as random slopes for individuals, for each age class separately. Best linear unbiased predictors (BLUPs) provided the slopes and intercepts needed to estimate individual mass on 15 August, as previously described (Kroeger et al., 2018; Maldonado‐Chaparro et al., 2015; Ozgul et al., 2010). To reduce the error created by extrapolation from small amounts of data, individuals with a single mass value, more than four weeks away from 15 August (i.e., before 15 July), were excluded from the dataset.

### 2.4 Over‐winter survival

Over‐winter survival was estimated either from recovery of a deceased individual (true survival) or from the absence/presence of the individual during the subsequent active season (apparent survival). In a previous study, using multi‐state mark‐recapture models, recapture probabilities of adults and juveniles were estimated to be higher than 0.90 in both sexes in all colonies (Ozgul et al., 2007). For yearlings, recapture probabilities ranged between 0.43 to 0.95 for females and between 0.56 and 1.00 for males depending on the colonies (Borrego et al., 2008; Ozgul et al., 2007). However, recapture probabilities of yearlings were estimated as >0.90 in all main colonies. Thus, all analyses were restricted to observations on animals from main colonies where survival is assumed to be known with a recapture rate >0.90 for all age classes. For yearling marmots, apparent annual survival from age 1 to age 2 may be confounded with natal dispersal. Dispersed individuals are not often recorded again within the study and dispersal is particularly high in male yearlings (Huang et al., 2011; Schwartz et al., 1998). While survival in these years cannot be perfectly disentangled from this additional factor, most dispersal occurs around the time of pup emergence. This can be controlled by excluding individuals who disappeared during this window (Montero et al., 2020); thus, using mass measurements on 15 August accounts for dispersal.

### 2.5 Statistical analyses

For mass‐survival analyses, we used data collected between 1962 and 2018. However, to control for the effects of selective disappearance, we kept only extinct or nearly extinct (<1% of individuals alive) cohorts and thus restricted the data to 1962–2015. The data collection for maximum running speed began in 2002 and continues to the present day. Consequently, when analyzing maximum running speed, we restricted all variables to data from 2002–2018. We ran four separate analyses for each question, based on the different life stages of yellow‐bellied marmots so that the final datasets consisted of: juveniles (number of individuals (*N*) and observations (*n*): *N*_survival_ = 1955, *N*_speed_ = 286, *n*_speed_ = 425), yearlings (*N*_survival_ = 955, *N*_speed_ = 124, *n*_speed_ = 185), subadults (*N*_survival_ = 324, *N*_speed_ = 27, *n*_speed_ = 48), and adults (*N*_survival_ = 234, *n*_survival_ = 687, *N*_speed_ = 56, *n*_speed_ = 124). All statistical analyses were done in R (R‐4.0.3, R Core Team, 2020). The packages lmerTest (v3.1.0, Kuznetsova et al., 2017) and lme4 (v1.1.21, Bates et al., 2015) were used to fit mixed‐effects models. Finally, all continuous independent variables were mean‐centered and scaled to a variance of 1 prior to analysis, allowing for comparison of coefficients across age classes.

To address the question of the impact of prehibernation body mass on over‐winter survival, we fitted generalized linear mixed models (glmer) with a binomial error structure and a logit link function. For each of the four age classes (i.e., juvenile, yearling, subadult, adult), sex, valley position, linear mass, and quadratic mass were included as fixed effects in the models, while colony and year of birth were included as random effects. For adults, since we had repeated measurements per individual, individual identity (ID) and observation year were also fitted as random effects. Linear and quadratic age were included as fixed effects in the adult model to take into account potential senescent effects. Additionally, an interaction between the two mass terms and valley or sex was tested for each age category and removed from the final model if insignificant, to avoid estimation biases (Engqvist, 2005). Significance of terms was evaluated based on the associated Z‐test comparing the estimated parameter to zero in the glmer output. To evaluate the significance of linear and quadratic effects of mass and age, orthogonal polynomials were used in all models. Orthogonal polynomials allow the linear and quadratic effects to be evaluated and tested independently, within a single model despite their collinearity (Kennedy, 1980; Narula, 1979). To allow for comparison with previous studies on selection, however, survival models were also re‐fitted to estimate selection gradients using raw polynomials, and only a linear effect of mass was fitted when the quadratic effect was not significant when using orthogonal polynomials.

To evaluate the link between body mass and running speed, we fitted linear mixed‐effects models of running speed for each age class separately. Sex, valley, substrate, and incline, in addition to linear and quadratic effects of mass, were included as fixed effects in all running speed models. Furthermore, we created an additional effect to account for the potential impact of habituation on running speed; trial number. Trial number was the cumulative total of trials undertaken by a particular individual, within the year of measurement. Additionally, for juveniles, growth is rapid and essentially linear until mid‐august leading to expected differences in cognitive and skeletal development, dependent on when running trials are conducted within the active season. Since not all litters emerge on the same date they cannot be considered at equivalent stages of development at set time points in the season, thus, age in days after emergence was also added in the model to control for between‐litter disparities. Age and quadratic age in years were also included in the model for adults to take into account potential impacts of senescence on running capabilities. Random effects included colony, birth year, individual identity, and observation year. Again, interactive terms of mass and valley, or sex, were tested for each age category, except the subadult age class where sample size was too small, and excluded when insignificant.

## 3 RESULTS

### 3.1 Effect of prehibernation body mass on over‐winter survival

**FIGURE 1:**
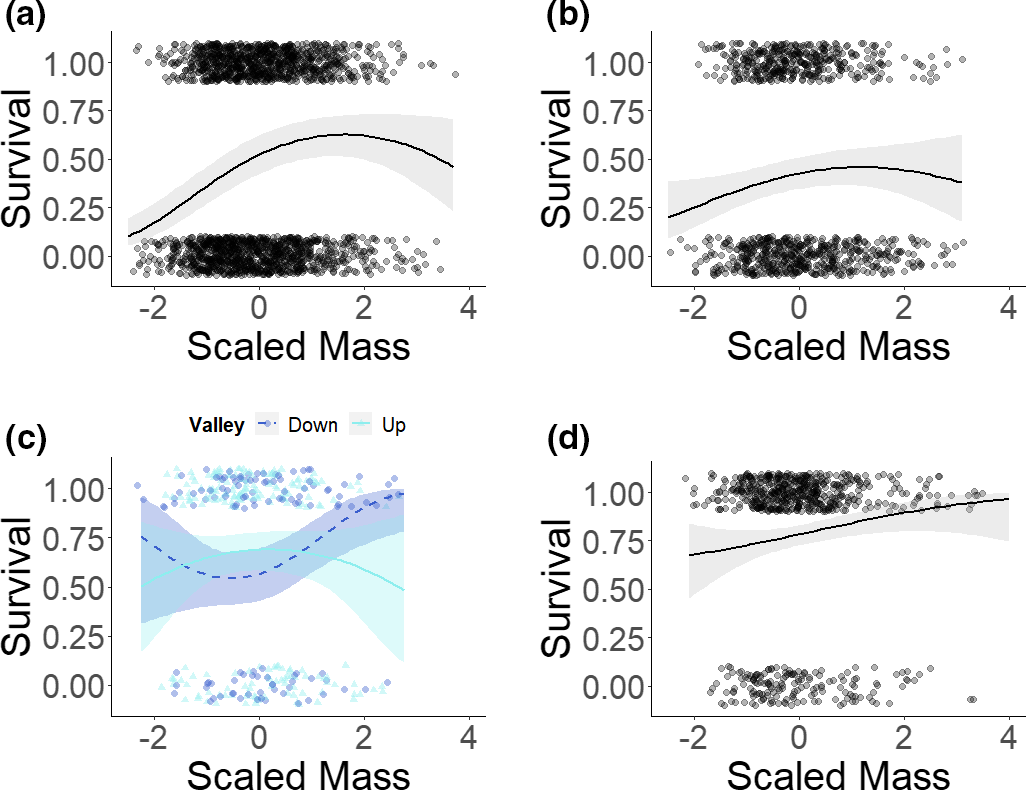
Relationships between prehibernation body mass (scaled mass) and probability of survival for (a) juvenile, (b) yearling, and (d) adult marmots. (c) shows the interaction of prehibernation body mass (scaled mass) and valley acting on probability of survival for subadult yellow‐bellied marmots living at high elevations (up‐valley) and at low elevations (down‐valley). In (c) low elevation predictions and data are indicated by dark blue color, circular points, and dashed lines whilst high elevations are indicated by pale blue coloration, triangular points, and solid lines. Body mass was scaled around the mean value for that age category to create scaled body mass. Points show the raw data, jittered to prevent data overlap. Black lines show the predicted models from Table S1 with 95% confidence intervals.

Results show that the shape and strength of viability selection on prehibernation body mass differed among age‐categories, moving from stabilizing selection in juveniles to positive directional selection in subadults and adults (Figure 1; Table 1; Table S1). In juvenile marmots, both the lightest and heaviest individuals had a reduced likelihood of surviving to the next active season when compared to individuals with intermediate prehibernation body mass (Figure 1a) whereas, in subadults and adults, selection on prehibernation body mass was more linear, with the heaviest individuals being more likely to survive over‐winter (Figure 1c,d). Yearling marmots (Figure 1b), also showed a weaker trend for over‐winter survival to increase with prehibernation body mass, however, the effect was nonsignificant. At all ages, marmot males were less likely to survive over‐winter, with the effect being significant from yearlings to adults (Table 1; Table S1).

**TABLE 1:** Selection gradients ± standard errors for generalized linear mixed models analyzing variation in annual survival at different life stages in yellow‐bellied marmots

Effect	Juvenile (0 years)	Yearling (1 years)	Subadult (2 years)	Adult (3+ years)
Intercept	−0.27 ± 0.27	0.19 ± 0.23	**0.73 ± 0.29**	**1.58 ± 0.19**
Mass	**0.51 ± 0.07**	0.17 ± 0.10	**0.34 ± 0.24**	**0.37 ± 0.16**
Mass^2^	**−0.16 ± 0.04**	—	0.32 ± 0.17	—
Sex (Male)	−0.16 ± 0.10	**−1.34 ± 0.16**	**−1.51 ± 0.35**	**−1.23 ± 0.42**
Valley (Up)	**0.87 ± 0.34**	0.15 ± 0.30	0.52 ± 0.35	−0.14 ± 0.42
Mass : Valley (Up)	—	—	−0.30 ± 0.28	—
Mass^2^ : Valley (Up)	—	—	**−0.45 ± 0.22**	—
Age in Years	—	—	—	**−0.08 ± 0.14**
Age^2^ in Years	—	—	—	**−0.15 ± 0.08**

Note

Random effects include colony, year of birth, individual identity (ID), and year of observation (year obs). Significant terms are in bold based on model using orthogonal polynomials reported in Table S1. When the quadratic term was not significant, the model for selection gradient fitted only a linear term. Terms not fitted are indicated with —.

The interaction between sex and prehibernation body mass was not significant at any life stage and, accordingly, was removed. In juveniles, individuals at higher elevations had higher survival probabilities (Table 1; Table S1). Within the subadult age class, an interaction between valley and the quadratic term for body mass (Figure 1c, Table 1; Table S1) showed that, for individuals at high elevations, there was little relationship between mass and survival. Whereas, for subadult individuals at low elevations, once individuals were larger than the mean prehibernation body mass, additional mass gains produced large advantages in terms of an increased chance of survival. Thus, directional selection is acting on the trait. Interestingly, an elevation‐dependent effect of body mass on survival was not observed at any other age class and so was excluded from all other models. Finally, in adults we found a quadratic relationship between chronological age and over‐winter survival, indicating that over‐winter survival declined as individuals got older (Table 1; Table S1). Furthermore, for adult models, the random effects of individual identity and colony prevented convergence and so were removed.

### 3.2 Body mass at trapping and maximum running speed

**FIGURE 2:**
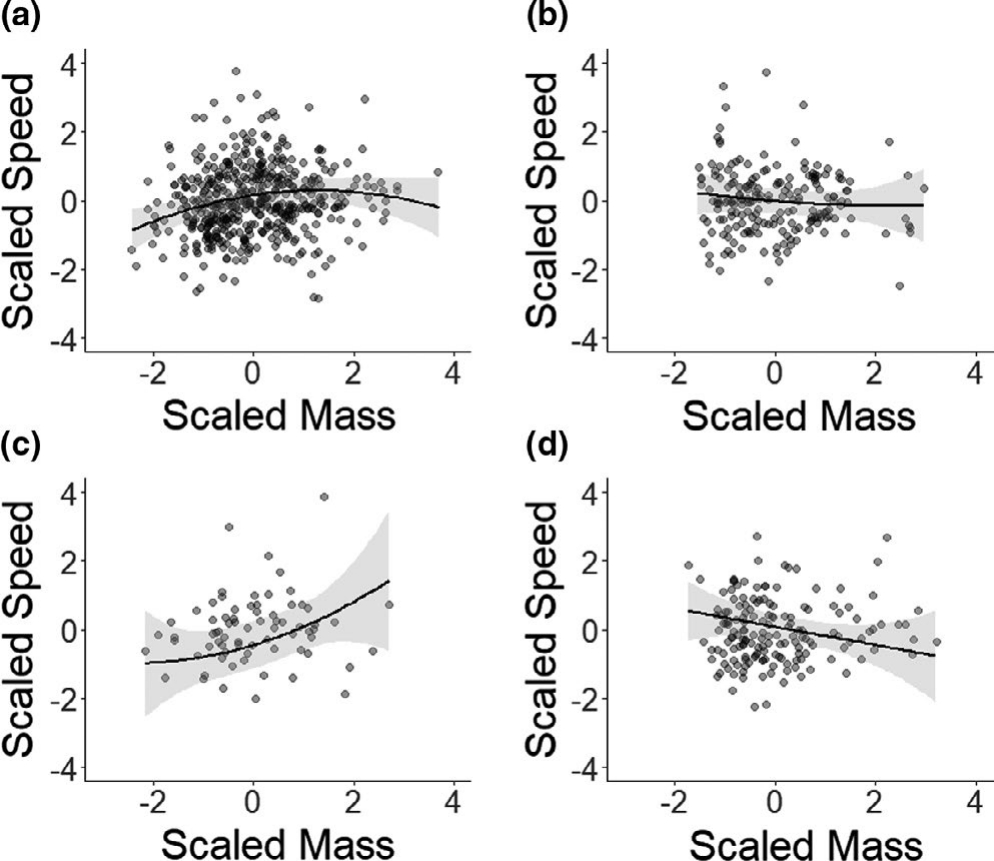
Relationships between body mass at trapping (scaled mass) and maximum running speed (scaled speed) for four age classes of yellow‐bellied marmot: juvenile (a), yearling (b), subadult, (c) and adult (d). Body mass was scaled around the mean premass at trapping, and maximum running speed around the mean speed at trapping, for that age category. Points show the raw data and black lines show the predicted models from Table 2 with 95% confidence intervals.

Similar to the observed associations between mass and survival, the relationship between body mass and speed varied depending on age class (Figure 2; Table 2; Table S2). Running speed was not significantly related to body mass in either yearling or adult models. However, for the juveniles, there were significant linear and quadratic effects of body mass (Figure 2a; Table 2; Table S2). The highest speeds were observed at intermediate‐mass, with animals at the lowest mass performing the worst and at the highest mass also showing slight decreases in speed. For subadults, there was a significant, positive linear relationship between maximum running speed whereby maximum running speed increased with linear body mass but not quadratic mass (Figure 2c Table 2; Table S2).

**TABLE 2:** Estimates ± standard errors for linear mixed‐effect models analyzing variation in maximum running speed at different life stages in yellow‐bellied marmots

Effect	Juvenile (0 years)	Yearling (1 years)	Subadult (2 years)	Adult (3+ years)
Intercept	**0.52 ± 0.20**	−0.07 ± 0.30	−1.27 ± 1.25	−0.01 ± 0.35
Mass	**3.88 ± 1.38**	−1.34 ± 1.54	**3.99 ± 1.80**	−3.35 ± 1.92
Mass^2^	**−2.56 ± 0.99**	0.44 ± 1.19	1.05 ± 1.71	−0.00 ± 1.18
Sex (Male)	−0.06 ± 0.09	**0.36 ± 0.16**	−0.75 ± 0.69	0.34 ± 0.47
Incline	−0.09 ± 0.05	−0.10 ± 0.08	−0.06 ± 0.15	0.01 ± 0.08
Substrate (High Veg)	**−0.70 ± 0.15**	−0.10 ± 0.25	0.39 ± 1.28	0.28 ± 0.35
Substrate (Low Veg)	**−0.29 ± 0.12**	0.04 ± 0.22	0.66 ± 1.20	**0.73 ± 0.32**
Substrate (Stone)	**−0.61 ± 0.18**	−0.22 ± 0.30	1.06 ± 1.08	0.09 ± 0.34
Valley (Up)	−0.20 ± 0.20	−0.06 ± 0.33	0.73 ± 0.54	**−0.65 ± 0.28**
Trial Number	0.04 ± 0.05	−0.05 ± 0.07	0.18 ± 0.14	−0.01 ± 0.084
Age in Days	−1.01 ± 0.70	—	—	—
Age in Years	—	—	—	−0.87 ± 1.25
Age^2^ in Years	—	—	—	−0.35 ± 1.22

Note

Linear and quadratic mass and age effects were fitted using orthogonal polynomials. Random effects include colony, year of birth, individual identity (ID), and year of observation (year obs). Significant terms are bolded (see Table S2 for details). Terms not fitted are indicated with —.

Effects of sex and trial conditions also differed across age‐categories. The effect of sex was not significantly different from zero for any age class except for yearlings, where males ran faster than females (Table 2; Table S2). Furthermore, substrate was only significant in models for juveniles and adults. In juveniles, marmots running over all substrates other than bare ground (dirt) were slower. However, in adults, the effect was reversed, with low vegetation significantly increasing speed and the high vegetation and stone substrates nonsignificantly increasing speed compared with dirt (Table 2; Table S2). The effect of elevation was significant only in adults, where individuals at higher elevation sites were slower than those at lower elevation sites (Table 2; Table S2). Incline and trial number did not affect maximum running speed in any age class nor did age in days or years exhibit significant effects in either the juvenile or adult models, respectively (Table 2; Table S2).

## 4 DISCUSSION

In this study, we showed that in yellow‐bellied marmot's patterns of viability selection on body mass, a key life‐history trait, varied across life stages and that the magnitude of the selective pressure acting on the trait differed across elevations. We also showed that the relationship between mass and maximum running speed varied across age classes. These results support two of our initial hypotheses: that the shape and strength of selection are stage‐dependent so that selection on body mass is principally directional and positive at older ages, but, is under stabilizing selection in juveniles; and, that a body mass—running speed trade‐off exists at young ages which could influence survival probability and be mediated by predation‐related selective pressure. However, we found no evidence for sex differences in selection on body mass for over‐winter survival. Altogether, our findings illustrate the importance of evaluating the requirements of different life stages when evaluating selection in long‐lived species.

Our results showed that prehibernation body mass, in the first year of life, is under stabilizing selection in yellow‐bellied marmots. This is in contrast with the expectation, for a hibernating species, of strong linear selection for larger individuals since winter energy requirements are supplied exclusively by fat reserves (Zervanos et al., 2014). There are multiple possible explanations for why hibernating juveniles might not benefit from larger body mass; limits on the availability of nutritional resources, diminishing returns from foraging (after some threshold size is achieved) or morphological constraints on the maximum possible energy store might all play a regulatory role (Humphries et al., 2003). A further explanation is the existence of a potential body mass—running speed trade‐off, as reported in other rodent species (Trombulak, 1989), impacting survival during encounters with predators. A large number of species prey on marmots; the main ones in our study system are coyotes (*Canis latrans*) and foxes (*Vulpes vulpes*). Vigilance and running speed are two key aspects of behavior employed to avoid predation from these species. Our results showed a trade‐off in juvenile marmots between running speed and body mass. In juvenile yellow‐bellied marmots, we propose that predation is one of the main drivers of stabilizing selection on prehibernation body mass. Within our system, juvenile marmots tend to be the last to immerse into hibernacula (Armitage, 2014), potentially because they have to gain enough energy reserves to sustain the demands of both skeletal growth and hibernation and they are vulnerable to predation later in the summer. Without the vigilance or warning calls provided by older members in the colony, and with the potential tendency for late‐season predation rates to be higher (reported in Vancouver Island marmots, *Marmota vancouverensis*, Bryant & Page, 2005), juveniles may be slower to detect predators and therefore more dependent on their own antipredatory behaviors, such as speed, to evade predation.

In both subadults and adults, animals with larger body masses, late in the season, have enhanced over‐winter survival. In yearlings, a tendency towards this trend was also reported. By observing the apparent directional selection on body mass within these groups, we might assume that hibernation is a key selective pressure regulating this trait from at least 2 years onwards. This is further supported by the absence of a trade‐off between mass and running speed within these age classes. We would, therefore, expect an increase in marmot body mass through time within this population as body mass and body condition are known to be heritable in this species (Martin *et al*., unpublished) as in many others (Mcadam et al., 2002; Merilä et al., 2001; Réale et al., 1999). However, the presence of viability selection in the earliest age class suggests that animals have already been subjected to 1 year of stabilizing selection and thus larger individuals have already been removed from the population before the age of recruitment, thereby diminishing the scope for selection. Furthermore, a previous study has shown that adult body mass has remained constant over the past decades (Ozgulet et al., 2010). In this study, by showing the existence of stabilizing viability selection at younger ages, we provide a potential mechanism for the “invisible fraction” bias (sensu Grafen, 1988) by which body mass at recruiting age might be constrained to lower values than might otherwise be predicted. Thus, stabilizing selection at earlier ages might be a reason for an apparent absence of response to selection when analyses are done only on adults.

Our findings also showed that subadults tend to have lower survival than adults, relative to values of body mass scaled about the mean. Subadults tend to be smaller than adults (Armitage, 2014) but are still capable of reproduction (Ozgul et al., 2007; Schwartz et al., 1998). It is possible that this reduction in survival reflects the cost of early reproduction and a subsequent inability, at young ages, to compensate for the resources lost in the rest of the active season whilst still growing. Additionally, within the subadult age class, we show that selection on body mass varies according to elevation. The reasoning behind this effect remains unclear since no site‐specific difference in the likelihood of reproducing or survival rate has been previously documented in our study population for this particular age class (Ozgul et al., 2006). However, recent work by Cordes et al. (2020) has indicated that marmots within our system, at 2 yrs or older, experience increased over‐winter survival when summers are wetter and snowmelt is later. Consequently, the elevational difference in selection patterns here may reflect a potential association between known elevational differences in environmental conditions (Blumstein, Im, et al., 2004), mass and survival that has yet to be investigated.

We also found that, within most age classes, males have consistently lower survival than females once corrected for body mass differences. There are several phenomena that could explain this pattern in yellow‐bellied marmots. Males in this species show higher levels of fecal glucocorticoid metabolites (Wey & Blumstein, 2012; Wey et al., 2015) and higher ectoparasitic burdens (Wey & Blumstein, 2012), indicating that male yellow‐bellied marmots are subject to more stressors. Additionally, given the expectation that, with increased body size, somatic structures must also scale up (McMahon, 1973), it is also possible that male marmots are in comparatively worse condition when compared with a female of the same mass. As mentioned before, yellow‐bellied marmots are sexually dimorphic (Armitage, 2014) and the males are expected to be structurally larger. Consequently, light male marmots may have proportionally smaller fat reserves than a similar size female with a smaller skeletomuscular structure. During our analyses we found however no evidence that marmot survival was dependent on the interplay between sex and body mass and, therefore, we provide no evidence that selection on mass differed between males and females as they mature (juvenile to yearling) and recruit into the population (yearling until adult).

We suggest that, given the apparent trade‐off between body mass and maximum running speed at younger ages, to understand the evolution of both body mass and antipredatory traits it is crucial to understand what is happening in all age classes. In addition, not acknowledging differences in selection across ages and forgetting that living adults have already survived multiple selection events can lead to incorrect predictions for evolution or population dynamics. In this species, assuming that mass is in fact strongly correlated across age classes, an observed absence of evolution in marmot adult mass despite positive selection on body mass or evolutionary stasis, could simply be due to the stabilizing selection on juveniles. Therefore, as our concluding message, we recommend that authors move away from examining selection on a phenotypic trait in only one age class or as a combination of all age classes together and expand their analyses to stage‐ or age‐specific selection analysis to better understand the selection and evolution of traits.

## CONFLICT OF INTEREST

None declared.

## AUTHOR CONTRIBUTIONS

**Alexandra H. M. Jebb:** Conceptualization (lead); Formal analysis (lead); Funding acquisition (equal); Investigation (lead); Methodology (lead); Visualization (lead); Writing‐original draft (lead); Writing‐review & editing (equal). **Daniel T. Blumstein:** Data curation (equal); Funding acquisition (equal); Project administration (lead); Writing‐review & editing (equal). **Julien G. A. Martin:** Conceptualization (equal); Data curation (equal); Formal analysis (supporting); Investigation (supporting); Methodology (supporting); Project administration (supporting); Supervision (equal); Validation (supporting); Visualization (supporting); Writing‐original draft (supporting); Writing‐review & editing (equal). **Pierre Bize:** Conceptualization (supporting); Formal analysis (supporting); Investigation (supporting); Methodology (supporting); Supervision (equal); Validation (supporting); Visualization (supporting); Writing‐original draft (supporting); Writing‐review & editing (equal).

### OPEN RESEARCH BADGES

This article has earned an Open Data Badge for making publicly available the digitally‐shareable data necessary to reproduce the reported results. The data is available at https://doi.org/10.17605/OSF.IO/CZTYF.

## Supporting information

Appendix S1

## Data Availability

Data is archived on OSF and available at https://doi.org/10.17605/OSF.IO/CZTYF (Identifier: https://doi.org/10.17605/OSF.IO/CZTYF).
