# Supplementary material for: Bigger is not always better: Viability selection on body mass varies across life stages in a hibernating mammal
Source: Ecol Evol. 2021 Mar 9;11(7):3435–45. doi: 10.1002/ece3.7304 (PMC8019046; doi:10.1002/ece3.7304)
Supplement: Supplementary file 1 — Appendix S1 [file ECE3-11-3435-s001.docx]

**Table S1,** Generalized linear mixed models of annual survival at different life stages in yellow-bellied marmots. Quantified fixed effects include the linear and quadratic effects of pre-hibernation body mass (using orthogonal polynomials, OP), sex (male or female), elevation inhabited (valley: up-valley or down-valley) as well as the linear and quadratic effects of age (years, with orthogonal polynomials) for adults only. Random effects include colony, year of birth, individual identity (ID) and year of observation (year obs). N_survival_ denotes the total number of individuals in each age category and n_survival_ the number of observations when repeated measures were taken per individual. Significant terms are bolded.

| **Effect** | **Estimate ± SE** | | | **z** | ***P*** |
| --- | --- | --- | --- | --- | --- |
| **Juvenile (0 yrs; N_survival_ = 1955)** |  |  |  |  |  |
| *V_year of birth_ = 0.58 (N = 48), V_colony_ = 0.10 (N = 4)* | | | | | |
| Intercept | -0.43 | ± | 0.27 | -1.59 | 0.111 |
| **OP Mass** | **19.44** | **±** | **3.14** | **6.18** | **< 0.001** |
| **OP Mass^2^** | **-9.54** | **±** | **2.38** | **-4.00** | **< 0.001** |
| Sex (Male) | -0.16 | ± | 0.10 | -1.58 | 0.113 |
| **Valley (Up)** | **0.87** | **±** | **0.34** | **2.53** | **0.011** |
| **Yearling (1 yrs; N_survival_ = 955)** |  |  |  |  |  |
| *V_year of birth_ = 0.23 (N = 51), V_colony_ = 0.06 (N = 4)* | | | | | |
| Intercept | 0.19 | ± | 0.23 | 0.23 | 0.41 |
| OP Mass | 5.17 | ± | 3.10 | 3.10 | 0.10 |
| OP Mass^2^ | -3.83 | ± | 2.38 | 2.38 | 0.11 |
| **Sex (Male)** | **-1.31** | **±** | **0.17** | 0.16 | **< 0.001** |
| Valley (Up) | 0.13 | ± | 0.30 | 0.30 | 0.67 |
| **Subadult (2 yrs; N_survival_ = 324)** |  |  |  |  |  |
| *V_year of birth_ = 0.30 (N = 49), V_colony_ = < 0.01 (N = 4)* | | | | | |
| **Intercept** | **1.05** | **±** | **0.25** | **4.17** | **< 0.001** |
| **OP Mass** | **8.49** | **±** | **4.30** | **1.98** | **0.048** |
| OP Mass^2^ | 7.58 | ± | 4.00 | 1.92 | 0.055 |
| **Sex (Male)** | **-1.51** | **±** | **0.35** | **-4.37** | **< 0.001** |
| Valley (Up) | 0.07 | ± | 0.27 | 0.25 | 0.804 |
| OP Mass : Valley (Up) | -8.55 | ± | 4.98 | -1.72 | 0.086 |
| **OP Mass^2^ : Valley (Up)** | **-10.68** | **±** | **5.10** | **-2.09** | **0.036** |
| **Adult (3+ yrs; N_survival_ = 234; n_survival_ = 687)** |  |  |  |  |  |
| *V_year obs_ = 0.07 (N = 54), V_year of birth_ = 0.08 (N = 49)* | | | | | |
| **Intercept** | **1.45** | **±** | **0.18** | **8.05** | **< 0.001** |
| **OP Mass** | **10.14** | **±** | **4.35** | **2.33** | **0.020** |
| OP Mass^2^ | 1.56 | ± | 2.75 | 0.57 | 0.571 |
| **Sex (Male)** | **-1.3** | **±** | **0.43** | **-3.01** | **0.003** |
| Valley (Up) | -0.15 | ± | 0.21 | -0.72 | 0.471 |
| **OP Age in Years** | **-6.78** | **±** | **2.67** | **-2.54** | **0.011** |
| **OP Age^2^ in Years** | **-4.78** | **±** | **2.44** | **-1.96** | **0.050** |

**Table S2,** Linear mixed-effect models of maximum running speed measured at different life stages in yellow-bellied marmots. Quantified fixed effects include the linear and quadratic effects of mass at the moment of trapping, sex (male or female), steepness of the path traversed (incline), terrain type (substrate: high vegetation, low vegetation, stone or dirt), elevation inhabited (valley: up-valley or down-valley), trial number, age in days (juveniles only), as well as the linear and quadratic effects of age (years) for adults only. For mass and age, linear and quadratic terms were fitted using orthogonal polynomials (OP). Random effects include colony, year of birth, individual identity (ID) and year of observation (year obs). The note n_speed_ displays the total number of observations when repeated measures were taken per individual. Significant terms are bolded.

| **Effect** | **Estimate ± SE** | | | **d.f.** | **t** | ***P*** |
| --- | --- | --- | --- | --- | --- | --- |
| **Juvenile (0 yrs; n_speed_ = 425)** |  |  |  |  |  |  |
| *V_ID_ = 0.00 (N = 286), V_year of birth_ = 0.17 (N = 13), V_colony_ = 0.07 (N = 12), V_residual_ = 0.72* | | | | | | |
| **Intercept** | **0.52** | **±** | **0.20** | **25.40** | **2.65** | **0.014** |
| **OP Mass** | **3.88** | **±** | **1.38** | **390.90** | **2.81** | **0.005** |
| **OP Mass^2^** | **-2.56** | **±** | **0.99** | **410.60** | **-2.59** | **0.010** |
| Sex (Male) | -0.06 | **±** | 0.09 | 411.51 | -0.69 | 0.489 |
| Incline | -0.09 | ± | 0.05 | 404.78 | -1.94 | 0.053 |
| **Substrate (High Veg)** | **-0.70** | **±** | **0.15** | 413.08 | -4.79 | **< 0.001** |
| **Substrate (Low Veg)** | **-0.29** | **±** | **0.12** | 400.35 | -2.34 | 0.020 |
| **Substrate (Stone)** | **-0.61** | **±** | **0.18** | 406.73 | -3.47 | **< 0.001** |
| Valley (Up) | -0.20 | **±** | 0.20 | 9.60 | -1.02 | 0.335 |
| Trial Number | 0.04 | **±** | 0.05 | 411.08 | 0.92 | 0.15 |
| Age in Days | -1.01 | **±** | 0.70 | 370.21 | -1.44 | 0.151 |
| **Yearling (1 yrs; n_speed_ = 185)** |  |  |  |  |  |  |
| *V_ID_ = 0.10 (N = 124), V_year of birth_ = 0.05 (N = 15), V_colony_ = 0.13 (N = 10), V_residual_ = 0.78* | | | | | | |
| Intercept | -0.07 | **±** | 0.30 | 11.30 | -0.24 | 0.817 |
| OP Mass | -1.34 | **±** | 1.54 | 15.19 | -0.87 | 0.396 |
| OP Mass**^2^** | 0.44 | **±** | 1.19 | 91.39 | 0.37 | 0.711 |
| **Sex (Male)** | **0.36** | **±** | **0.16** | **121.47** | **2.22** | **0.028** |
| Incline | -0.10 | **±** | 0.08 | 174.15 | -1.35 | 0.180 |
| Substrate (High Veg) | -0.10 | **±** | 0.25 | 164.67 | -0.39 | 0.694 |
| Substrate (Low Veg) | 0.04 | **±** | 0.22 | 170.36 | 0.18 | 0.857 |
| Substrate (Stone) | -0.22 | **±** | 0.30 | 169.27 | -0.72 | 0.473 |
| Valley (Up) | -0.06 | **±** | 0.33 | 7.23 | -0.19 | 0.852 |
| Trial Number | -0.05 | **±** | 0.07 | 167.57 | -0.62 | 0.535 |
| **Subadult (2 yrs; n_speed_ = 48)** |  |  |  |  |  |  |
| *V_ID_ = 0.015 (N = 27), V_year of birth_ = 0.04 (N = 12), V_colony_ = 0.23 (N = 8), V_residual_ = 0.91* | | | | | | |
| Intercept | -1.27 | **±** | 1.25 | 27.59 | -1.02 | 0.316 |
| **OP Mass** | **3.99** | **±** | **1.80** | **15.10** | **2.21** | **0.043** |
| OP Mass**^2^** | 1.05 | **±** | 1.71 | 25.30 | 0.61 | 0.547 |
| Sex (Male) | -0.75 | **±** | 0.69 | 30.86 | -1.09 | 0.283 |
| Incline | -0.06 | **±** | 0.15 | 34.21 | -0.37 | 0.713 |
| Substrate (High Veg) | 0.39 | **±** | 1.28 | 27.74 | 0.30 | 0.764 |
| Substrate (Low Veg) | 0.66 | **±** | 1.20 | 26.94 | 0.55 | 0.587 |
| Substrate (Stone) | 1.06 | **±** | 1.08 | 18.04 | 0.98 | 0.340 |
| Valley (Up) | 0.73 | **±** | 0.54 | 2.74 | 1.34 | 0.282 |
| Trial Number | 0.18 | **±** | 0.14 | 35.62 | 1.27 | 0.212 |
| **Adult (3+ yrs; n_speed_ = 124)** |  |  |  |  |  |  |
| *V_ID_ = 0.31 (N = 56), V_year obs_ = 0.03 (N = 15), V_year of birth_ = 0.00 (N = 21), V_colony_ = 0.03 (N = 11), V_residual_ = 0.49* | | | | | | |
| Intercept | -0.01 | **±** | 0.35 | 37.82 | -0.02 | 0.986 |
| OP Mass | -3.35 | **±** | 1.92 | 84.20 | -1.74 | 0.085 |
| OP Mass**^2^** | -0.00 | **±** | 1.18 | 72.54 | 0.00 | 0.998 |
| Sex (Male) | 0.34 | **±** | 0.47 | 82.15 | 0.72 | 0.472 |
| Incline | 0.01 | **±** | 0.08 | 102.71 | 0.09 | 0.929 |
| Substrate (High Veg) | 0.28 | **±** | 0.35 | 102.83 | 0.82 | 0.413 |
| **Substrate (Low Veg)** | **0.73** | **±** | **0.32** | **102.94** | **2.31** | **0.023** |
| Substrate (Stone) | 0.09 | **±** | 0.34 | 95.54 | 0.27 | 0.788 |
| **Valley (Up)** | **-0.65** | **±** | **0.28** | **8.33** | **-2.37** | **0.044** |
| Trial Number | -0.01 | ± | 0.084 | 94.90 | -0.14 | 0.886 |
| OP Age in Years | -0.87 | **±** | 1.25 | 53.08 | -0.69 | 0.492 |
| OP Age**^2^** in Years | -0.35 | **±** | 1.22 | 53.07 | -0.28 | 0.777 |

**Appended Analysis,** For an additional analysis run on a subset of data, we fitted linear mixed effects evaluating the link between body mass and maximum running speed for each age class defined in the main body of the text (juvenile, yearling, subadult, adult). We followed the same procedure as stated in the methods section of this paper; fitting sex, valley, substrate, incline, trial number, age (in days for juveniles and in years for adults) as well as linear and quadratic effects of mass as fixed effects. Random effects were fitted as colony, year of birth, individual identity and year of observation. Interactions of mass by valley or mass by sex were tested and excluded when insignificant, except for within the subadult age class where we lacked data to evaluate these terms. All continuous variables were mean-centred and scaled to a variance of 1. The data within each age category was reduced so that all short runs (<1.5 s) were excluded. This resulted in a final reduction of the number of observations (juvenile: - 125, yearlings: - 38, subadult: - 8, adult: - 20) and number of individuals included in the analysis (juvenile: - 76, yearlings: - 22, subadult: - 3, adult: - 8).

We obtained quantitatively similar results from these models to those in the main body of the paper (Appendix Table 2). Within the juvenile age class, the significant effects of the quadratic effect of body mass on maximum running speed as well as the negative impact of high vegetation and stone were all reduced (Appendix Table 3).

Despite these results, and some potential associated error, we focus on discussing the models including measurements under the 1.5 s margin as we believe them to be of biological significance and indicative of the weakest/poorest runners within the population.

**Table S3,** Linear mixed-effect models of maximum running speed measured at different life stages in yellow-bellied marmots when run time was > 1.5 s. Quantified fixed effects include the linear and quadratic effects of mass at the moment of trapping, sex (male or female), steepness of the path traversed (incline), terrain type (substrate: high vegetation, low vegetation, stone or dirt), elevation inhabited (valley: up-valley or down-valley), trial number, age in days (juveniles only), as well as the linear and quadratic effects of age (years) for adults only. For mass and age, linear and quadratic terms were fitted using orthogonal polynomials (OP). Random effects include colony, year of birth, individual identity (ID) and year of observation (year obs). The note n_speed_ displays the total number of observations when repeated measures were taken per individual. Significant terms are bolded.

| **Effect** | **Estimate ± SE** | | | **d.f.** | **t** | ***P*** |
| --- | --- | --- | --- | --- | --- | --- |
| **Juvenile (0 yrs; n_speed_ = 300)** |  |  |  |  |  |  |
| *V_ID_ = 0.00 (N = 210), V_year of birth_ = 0.18 (N = 13), V_colony_ = 0.12 (N = 12), V_residual_ = 0.65* | | | | | | |
| Intercept | 0.37 | ± | 0.24 | 27.10 | 1.53 | 0.137 |
| **OP Mass** | **2.67** | **±** | **1.34** | **275.44** | **1.99** | **0.047** |
| OP Mass^2^ | -1.49 | ± | 0.96 | 285.36 | -1.56 | 0.121 |
| Sex (Male) | 0.10 | ± | 0.10 | 285.37 | 0.94 | 0.347 |
| Incline | -0.06 | ± | 0.05 | 285.75 | -1.08 | 0.281 |
| **Substrate (High Veg)** | **-0.68** | **±** | **0.17** | **287.27** | **-4.00** | **< 0.001** |
| Substrate (Low Veg) | -0.26 | ± | 0.15 | 283.22 | -1.74 | 0.082 |
| Substrate (Stone) | -0.24 | ± | 0.21 | 279.02 | -1.14 | 0.256 |
| Valley (Up) | -0.25 | ± | 0.25 | 10.87 | -1.00 | 0.341 |
| Trial Number | 0.04 | ± | 0.05 | 284.33 | 0.73 | 0.465 |
| Age in Days | -0.03 | ± | 0.08 | 256.41 | -0.40 | 0.69 |
| **Yearling (1 yrs; n_speed_ = 147)** |  |  |  |  |  |  |
| *V_ID_ = 0.15 (N = 102), V_year of birth_ = 0.09 (N = 14), V_colony_ = 0.14 (N = 10), V_residual_ = 0.66* | | | | | | |
| Intercept | -0.09 | ± | 0.33 | 14.88 | -0.28 | 0.787 |
| OP Mass | -2.21 | ± | 1.62 | 24.60 | -1.36 | 0.186 |
| OP Mass^2^ | 0.23 | ± | 1.19 | 88.92 | 0.19 | 0.851 |
| **Sex (Male)** | **0.38** | **±** | **0.18** | **102.97** | **2.18** | **0.032** |
| Incline | -0.11 | ± | 0.08 | 136.73 | -1.33 | 0.185 |
| Substrate (High Veg) | 0.07 | ± | 0.28 | 126.09 | 0.25 | 0.803 |
| Substrate (Low Veg) | 0.13 | ± | 0.24 | 130.27 | 0.55 | 0.585 |
| Substrate (Stone) | -0.26 | ± | 0.33 | 135.65 | -0.79 | 0.431 |
| Trial Number | -0.01 | ± | 0.08 | 134.58 | -0.16 | 0.872 |
| Valley (Up) | -0.11 | ± | 0.35 | 7.59 | -0.30 | 0.770 |
| **Subadult (2 yrs; n_speed_ = 40)** |  |  |  |  |  |  |
| *V_ID_ = 0.00 (N = 24), V_year of birth_ = 0.14 (N = 10), V_colony_ = 0.00 (N = 8), V_residual_ = 1.02* | | | | | | |
| Intercept | -0.74 | ± | 1.32 | 29.94 | -0.57 | 0.576 |
| **OP Mass** | **5.26** | **±** | **1.92** | **19.19** | **2.74** | **0.013** |
| OP Mass^2^ | 0.74 | ± | 1.75 | 29.37 | 0.42 | 0.676 |
| Sex (Male) | -1.92 | ± | 0.95 | 29.20 | -2.03 | 0.051 |
| Incline | -0.15 | ± | 0.18 | 29.01 | -0.83 | 0.414 |
| Substrate (High Veg) | -0.01 | ± | 1.38 | 27.52 | -0.01 | 0.994 |
| Substrate (Low Veg) | 0.31 | ± | 1.30 | 29.09 | 0.24 | 0.811 |
| Substrate (Stone) | 0.78 | ± | 1.25 | 25.97 | 0.62 | 0.541 |
| Trial Number | 0.30 | ± | 0.16 | 30.00 | 1.87 | 0.072 |
| Valley (Up) | 0.61 | ± | 0.42 | 19.76 | 1.44 | 0.166 |
| **Adult (3+ yrs; n_speed_ = 104)** |  |  |  |  |  |  |
| *V_ID_ = 0.20 (N = 48), V_year obs_ = 0.05 (N = 15), V_year of birth_ = 0.00 (N = 19), V_colony_ = 0.15 (N = 11), V_residual_ = 0.42* | | | | | | |
| Intercept | -0.29 | ± | 0.43 | 22.63 | -0.68 | 0.503 |
| **OP Mass** | **-5.65** | **±** | **1.93** | **68.23** | **-2.92** | **0.005** |
| OP Mass^2^ | 0.59 | ± | 1.08 | 65.94 | 0.55 | 0.586 |
| Sex (Male) | 0.72 | ± | 0.45 | 58.83 | 1.61 | 0.113 |
| Incline | 0.07 | ± | 0.08 | 85.45 | 0.88 | 0.382 |
| Substrate (High Veg) | 0.43 | ± | 0.35 | 82.89 | 1.22 | 0.227 |
| **Substrate (Low Veg)** | **0.96** | **±** | **0.33** | **85.80** | **2.92** | **0.004** |
| Substrate (Stone) | 0.05 | ± | 0.36 | 78.71 | 0.15 | 0.880 |
| Valley (Up) | -0.54 | ± | 0.37 | 6.13 | -1.47 | 0.191 |
| Trial Number | -0.02 | ± | 0.09 | 84.41 | -0.25 | 0.807 |
| OP Age in Years | -0.65 | ± | 1.10 | 48.37 | -0.59 | 0.558 |
| OP Age^2^ in Years | -1.15 | ± | 1.08 | 40.77 | -1.06 | 0.294 |
